# *μ*-CS: An extension of the TM4 platform to manage Affymetrix binary data

**DOI:** 10.1186/1471-2105-11-315

**Published:** 2010-06-10

**Authors:** Pietro H Guzzi, Mario Cannataro

**Affiliations:** 1 Bioinformatics Laboratory, Department of Experimental Medicine and Clinic, Magna Graecia University, Catanzaro, Italy; 2 ICAR, CNR, Rende, Italy

## Abstract

**Background:**

A main goal in understanding cell mechanisms is to explain the relationship among genes and related molecular processes through the combined use of technological platforms and bioinformatics analysis. High throughput platforms, such as microarrays, enable the investigation of the whole genome in a single experiment. There exist different kind of microarray platforms, that produce different types of binary data (images and raw data). Moreover, also considering a single vendor, different chips are available. The analysis of microarray data requires an initial preprocessing phase (i.e. normalization and summarization) of raw data that makes them suitable for use on existing platforms, such as the TIGR M4 Suite. Nevertheless, the annotations of data with additional information such as gene function, is needed to perform more powerful analysis. Raw data preprocessing and annotation is often performed in a manual and error prone way. Moreover, many available preprocessing tools do not support annotation. Thus novel, platform independent, and possibly open source tools enabling the semi-automatic preprocessing and annotation of microarray data are needed.

**Results:**

The paper presents *μ*-CS (Microarray Cel file Summarizer), a cross-platform tool for the automatic normalization, summarization and annotation of Affymetrix binary data. *μ*-CS is based on a client-server architecture. The *μ*-CS client is provided both as a plug-in of the TIGR M4 platform and as a Java standalone tool and enables users to read, preprocess and analyse binary microarray data, avoiding the manual invocation of external tools (e.g. the Affymetrix Power Tools), the manual loading of preprocessing libraries, and the management of intermediate files. The *μ*-CS server automatically updates the references to the summarization and annotation libraries that are provided to the *μ*-CS client before the preprocessing. The *μ*-CS server is based on the web services technology and can be easily extended to support more microarray vendors (e.g. Illumina).

**Conclusions:**

Thus *μ*-CS users can directly manage binary data without worrying about locating and invoking the proper preprocessing tools and chip-specific libraries. Moreover, users of the *μ*-CS plugin for TM4 can manage Affymetrix binary files without using external tools, such as APT (Affymetrix Power Tools) and related libraries. Consequently, *μ*-CS offers four main advantages: (i) it avoids to waste time for searching the correct libraries, (ii) it reduces possible errors in the preprocessing and further analysis phases, e.g. due to the incorrect choice of parameters or the use of old libraries, (iii) it implements the annotation of preprocessed data, and finally, (iv) it may enhance the quality of further analysis since it provides the most updated annotation libraries. The *μ*-CS client is freely available as a plugin of the TM4 platform as well as a standalone application at the project web site (http://bioingegneria.unicz.it/M-CS).

## Background

A main objective of functional genomics is to understand the relationship among genes and molecular functions. Researchers try to elucidate these relations through the systematic investigation of genes activity, for instance by using the microarray technique that enables the monitoring of all the genes during the cell phases or during the response to an external stimuli [1]. The central dogma of molecular biology merges together DNA, RNA, and proteins in a close relation, so the investigation of the RNA elucidates the functions of DNA. DNA microarrays enable the investigation of the activity of genes in different conditions (e.g. in different temporal points or under different drug concentrations). Recent microarray chips, such as the Affymetrix [2] Human Gene 1.0 ST, enable the simultaneous investigation of more than 33.000 genes. Such technology uses a single chip to monitor the activity of a set of genes through the investigation of the mRNA. Each chip uses a large number of probes to bind the mRNA of the biological sample under investigation. Each probe is marked with a fluorescent colour, so the fluorescence intensity takes into account the amount of the mRNA present in the sample. Usually, microarrays employ a redundant number of probes in order to minimise the experimental error in intensity measurement. Thus the value of fluorescence intensity associated to each gene has to be deducted from the values of all the associated probes.

The first output of a microarray experiment is an image where pixels intensities are related to gene expressions values. Images are then encoded into numerical data by using proprietary tools that extract regions corresponding to probes and convert their pixel intensities into a numerical value. These files usually use a proprietary format defined by the microarray vendors and are not automatically readable by existing analysis platforms, thus the analysis of microarray data requires a preliminary preprocessing activity before the further analysis [3-7]. The preprocessing involves several phases, among those: denoising, background correction, normalization and summarization, i.e. the computation of a specific gene expression value obtained by combining the values of corresponding probes [8,9].

For instance, let us consider the analysis of microarray data derived by Affymetrix chips with the TM4 [10] platform. This platform is not able to directly manage the Affymetrix CEL files, so the user has to perform some steps manually employing external tools such as the Affymetrix Expression console [2] or the Affymetrix Power Tools [2], or third part software such as RMAExpress [11]. The process starts with the summarization phase, i.e. converting images into numerical data. This phase combines multiple probe intensities into a single expression value. All arrays employ more than one probe for each gene as introduced before. Summarization takes into account all of the probes for the same gene and averages them enhancing the signal-to-noise ratio. Summarization requires the use of proper libraries that store the association among pixels and probesets. Such libraries are provided by Affymetrix as Chip Description File (CDF).

For Affymetrix arrays, summarization is usually done together with the normalization, using the same Affymetrix libraries which store the topological information about probes. There exist different summarization algorithms for expression arrays, such as the Robust Multi-array Average (RMA) [11] and the Probe Logarithmic Intensity Error (PLIER) [12], included into the Affymetrix tools. Moreover, for exon arrays, the Detection Above BackGround (DABG) [13] method is also used to generate a detection metric to enhance the detection of background noise.

The simplest approach for normalizing microarray data is to re-scale each expression value of a dataset. There exist two main approaches for normalization: the quantile algorithm [14] and the sketch-quantile algorithm that requires less computational resources.

For instance, the following command line shows the use of APT to normalize and summarize an Affymetrix dataset:

apt-probeset-summarize -a rma -d HuEx-1_0-st-v2.cdf -o/home/output -cel-files/home/list.txt

In particular, the rma option specifies the RMA summarization algorithm, the -d HuEx-1_0-st-v2.cdf option specifies the Human Exon 1.0 st library, while the -o -cel-files options specify respectively the output and input folders (where list.txt specifies the list of input cel files).

After summarization and normalization, the user has to associate to each expression value the related gene and eventually some further biological annotation, such as the gene symbol or information extracted by Gene Ontology [15]. Often annotation files are provided by the chip manufacturer and contain different levels of annotation, e.g. database identifier, description of molecular function, associated protein domains. It should be noted that not all the preprocessing tools allow the annotation of gene expression values. Finally, preprocessed data, organized in a suitable data structure (e.g. a comma separated value table), can be read and analyzed by the TM4 platform. The main drawbacks of such an approach are: (i) the need to generate and store intermediate files in a manual way that prevent the automation of the process; (ii) the need to know the details of the used chips and related preprocessing tools and libraries; (iii) the need to manually download the most updated summarization and annotation libraries from the vendor website, and (iv) the need to manually import preprocessed files in the analysis platforms, that may introduce errors. The automation of the preprocessing pipeline could speed-up the entire analysis process and reduce possible errors, allowing the user to concentrate on biological aspects. From this scenario, in order to enable the sharing and cross-comparison of microarray results from different platforms and laboratories, we propose a software tool to automatize the preprocessing of raw microarray data.

The proposed tool, named *μ*-CS, is able to preprocess Affymetrix microarray data in order to simplify and automatize the summarization, normalization, and annotation of microarray data. *μ*-CS is based on a distributed architecture and uses the client/server model. The *μ*-CS server is in charge of tracing the versions of libraries used to preprocess arrays data and made available by microarray vendors, allowing a transparent access to the right and most updated versions of preprocessing libraries. The *μ*-CS client implements the preprocessing methods by wrapping the Affymetrix APT preprocessing tools and implements the annotation process by joining summarized data with annotation libraries. The main idea of *μ*-CS is to reduce the number of information provided by the user during the data preprocessing, by embedding chip and software details into the *μ*-CS databases. Examples of such details are the chip type and version, the software version, the link where to download the last version of the Affymetrix Power Tools and annotation libraries, what chip library and annotation library need to be used for a given chip, and so on.

## Implementation

**Figure 1:**
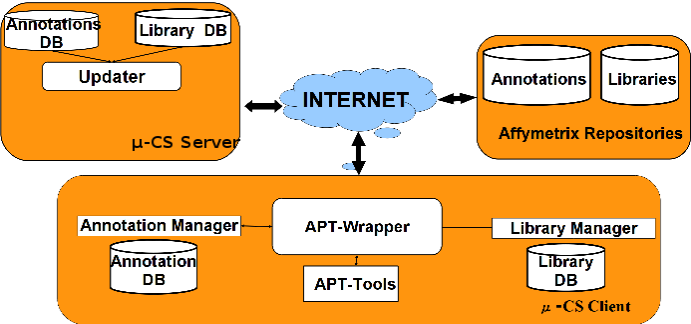
**Architecture of *μ*-CS**. Figure depicts the distributed architecture of *μ*-CS

The *μ*-CS tool adopts a client/server architecture as depicted in Figure 1: the *μ*-CS client wraps the APT preprocessing tools and offers the summarization and annotation functions to the user through an intuitive user interface, while the *μ*-CS server maintains the association between chip type and summarization/annotation libraries and an updated list of pointers (i.e. URLs) to the last available versions of such libraries. The *μ*-CS client is implemented both as a standalone tool and as a plugin for the TM4 platform.

The *μ*-CS system has been implemented using different techniques: (i) the standalone and the plugin versions of the client are implemented as Java [16] desktop applications that wrap the APT executable through the runtime support of Java, (ii) the server is implemented by using the Web Services [17] technology and the PHP [18] language and has been deployed on an Apache web server, (iii) the client communicates with the server using standard SOAP messages over HTTP protocol, (iv) the server communicates with the Affymetrix [2] repositories through HTTP messages.

## The *μ*-CS client

The *μ*-CS client offers to the user the normalization, summarization and annotation of microarray data. The plugin version, embedded into TM4, also offers the analysis features of TM4, while the standalone version makes available the preprocessed and annotated data for further analysis.

The *μ*-CS client (see Figure 1) comprises the following modules: (*i*) the **APT Wrapper**, (*ii*) the **Library Manager**, and (*iii*) the **Annotation Manager**. The Library Manager and the Annotation Manager store, respectively, the summarization and annotation libraries on two databases named **LibraryDB **and **AnnotationDB**. The APT Wrapper is able to invoke the Affymetrix Power Tools executable without user intervention by using the libraries mentioned above. Moreover, it includes a procedure to join the summarized data with the annotations contained in the AnnotationDB.

**Figure 2:**
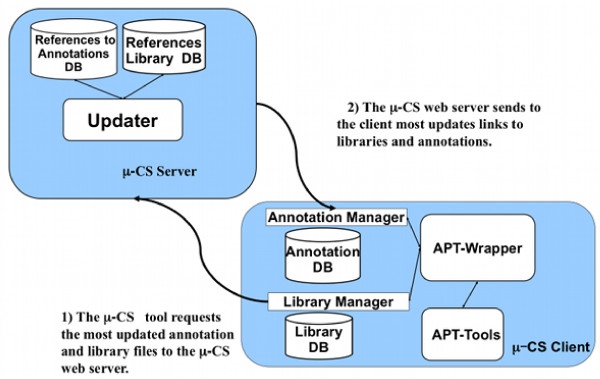
**The Update of the Client Databases**. Figure depicts the process of updating of the client database.

A TM4 user needing to analyze a CEL files dataset launches the *μ*-CS client plugin version. The *μ*-CS client asks to the *μ*-CS server for the last available libraries and obtains a reference (i.e. URLs) to them. Then, it downloads the needed libraries and stores them into the local databases as depicted in Figure 2. The communication among server and client is based on the exchange of an XML file containing the URLs of the libraries. Finally, the APT Wrapper invokes the APT executable by providing the right summarization library contained in LibraryDB. An instance of APT is invoked whenever a preprocessing request is received. After the normalization and summarization job is completed, the APT wrapper generates the preprocessed data stored in a comma separated values table. If annotations are available, the *μ*-CS client annotates the data by using the right annotation library contained in AnnotationDB.

## The *μ*-CS server

**Figure 3:**
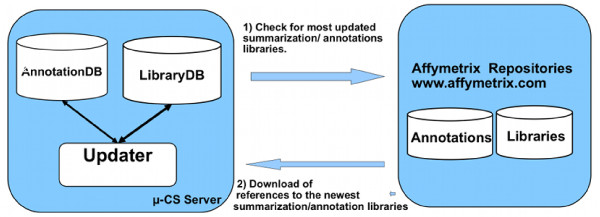
**The Update of the Server Databases**. The process of updating references to the newest libraries and annotation that involves the *μ*-CS and the Affymetrix server

The *μ*-CS server implements a repository of medatata about the type of chips, the preprocessing and annotation libraries and their location inside the Affymetrix repository. The goal of the *μ*-CS server is to provide to the *μ*-CS client an updated version of summarization and annotation libraries that periodically can be updated by the vendors, hiding to the users the details of such data updates as depicted in Figure 3. The *μ*-CS server comprises the following modules: the **Updater Web Service**, the **LibraryDB **and the **AnnotationDB. **The LibraryDB and AnnotationDB contain references (i.e. URLs) to the summarization and annotation libraries stored in the Affymetrix repository. The Updater Web Service, implemented as a Web Service [17], periodically (daily) verifies the existence of newest version of libraries and annotations by connecting to the Affymetrix repository. If it finds available updates for the LibraryDB or for the AnnotationDB, then it downloads the references to these updates and stores them into the AnnotationDB and LibraryDB.

The rest of the Section shows the functionalities of *μ*-CS through a case study on Affymetrix binary files: the analysis of a Human Gene 1.0 dataset freely available for download on the Affymetrix web site [2]. This dataset contains various mixture levels of two tissues: brain and heart from Human samples. We selected 10 arrays from these to perform our study.

The following paragraphs describe the step-by-step use of the *μ*-CS client (plugin version). The functions of the plugin and standalone versions are identical. They differ only in the following: the former is launched from the TM4 menu and then preprocessed data are directly available for analysis in TM4, while the latter is launched as an autonomous tool and then preprocessed data are available for different analysis platforms.

## Step 1: Installing/updating libraries

**Figure 4:**
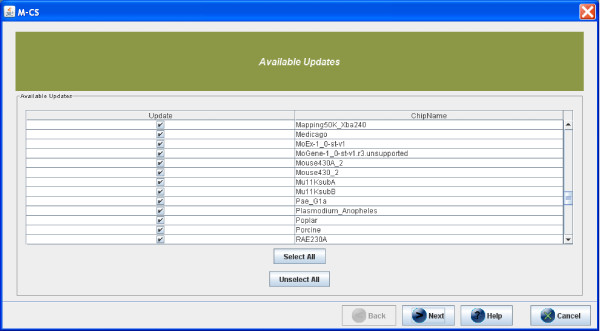
**Choice of libraries of *μ*-CS**. The GUI of *μ*-CS lists all the available summarization and annotation libraries.

After the generation of raw Affymetrix microarray data, the main preprocessing steps are: (i) normalization, (ii) summarization, and (iii) annotation. Normalization consists of reducing the bias among chips and within different regions of the same chip, aiming at removing non-biological variability within a dataset. Summarization combines multiple preprocessed probe intensities to a single expression value. Annotation associates to each probe its known annotations such as Gene Symbol or Gene Ontology. Each step of preprocessing requires proper tools and libraries that are provided by Affymetrix and that are periodically updated. The *μ*-CS client maintains a list of libraries that are shown to the user through the GUI (Graphical User Interface) as depicted in Figure 4.

**Figure 5:**
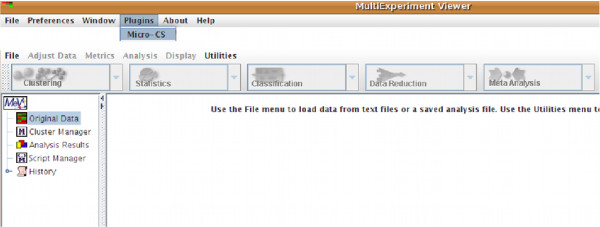
Launch of *μ*-CS from TMeV

**Figure 6:**
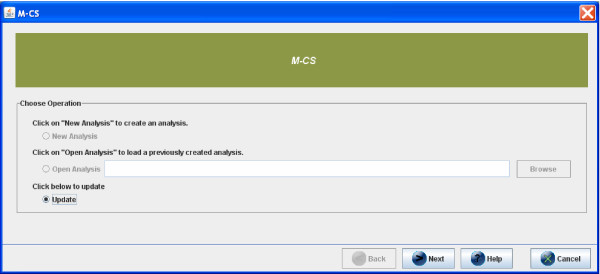
**Installing Libraries in *μ*-CS**. Installation of libraries in *μ*-CS.

After launching the TM4 platform (which includes the *μ*-CS plugin) the user can invoke the plugin from the TM4 toolbar menu, as depicted in Figure 5. As depicted in Figure 6, through the GUI of *μ*-CS the user can: (i) load a new CEL files dataset (New Analysis option), (ii) load a previously used dataset (Open Analysis option), or (iii) downloading or updating libraries (Update Option).

This last step can be skipped if libraries are already installed, or if the user does not want to update them. Alternatively the user has to install the libraries as described in the following. Nevertheless, the first time when *μ*-CS is used, the user has to install the needed libraries as depicted, while the subsequent times *μ*-CS checks only for available updates.

The *μ*-CS client queries the *μ*-CS server for the needed libraries and receives the references to the updated libraries so it can download and install them. After the first use, the *μ*-CS client stores the last used libraries in its local databases. Then the *μ*-CS client automatically presents to the user the installed libraries that are up to date with the vendor repository. User can select libraries to be installed by simply point and click on the chip name, as depicted in Figure 4.

## Step 2: Loading Dataset and Selection of Chip Libraries

First of all the user has to select the proper chip type, e.g. Human Gene 1.0 ST, that also identifies the library. In fact, the choice of the chip determines what preprocessing executable need to be invoked and what summarization and annotation libraries are needed. The selection of chip type and version is performed through the interface. Then the user has to select and load into the *μ*-CS client the raw microarray data files forming the dataset to be preprocessed (i.e. a set of CEL files).

## Step 3: APT parameters setting

**Figure 7:**
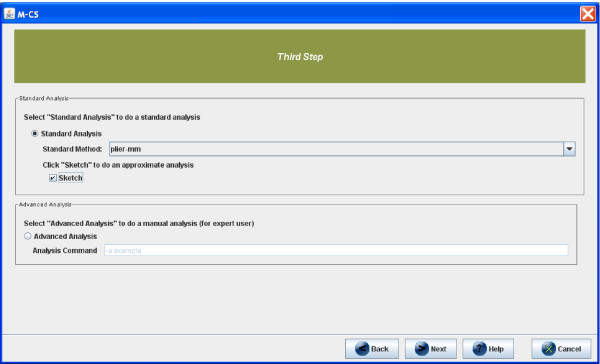
**Setting preprocessing parameter in *μ*-CS**. Figure depicts the choice of Plier as summarization method and sketch as normalization scheme

After obtaining the updated libraries, the user has to set the parameters of the APT preprocessing executable. In particular, *μ*-CS allows to use different parameter settings of APT to allow a flexible use to different categories of users. By selecting the *Standard Analysis *mode, most of the users may set the most important parameters, while in *Advanced Analysis *mode advanced users may explicitly set all APT parameters. User can choose to perform a *Standard Analysis *or an *Advanced Analysis *(see Figure 7). In the first case, the *μ*-CS client, as depicted in Figure 7, permits the choice of the summarization algorithm (RMA, PLIER, DABG) and the normalization type (the default quantile normalization or the faster sketch-quantile one). In particular, in Figure 7, the following parameters have been selected: Summarization Methods = Plier and Sketch = Yes. In *Advanced Analysis *mode, the user can set manually all the parameters and options of APT. For instance, if the user inserts the following APT parameters in the *μ*-CS GUI: -a rma -a plier - mm sketch -o chip.pgf -c chip.clf/home/hiram/output *.cel, the following command line will be generated apt-probeset-summarize -a rma -a plier - mm sketch -o chip.pgf -c chip.clf/home/hiram/output *.cel.

## Step 4: Preprocessing and annotation

After selecting the input dataset, the chip type and libraries, and the APT parameters, the *μ*-CS client proceeds with the preprocessing and annotation of the data, by invoking the APT executable with the user's specified parameters. Different procedures are necessary if the user want to conduct a gene level or an exon level analysis. In fact for exon level analysis the user can use two times the *μ*-CS, the first time to normalize and summarize data and the second one to find the background noise trough DABG. When summarization is completed, the *μ*-CS client annotates the results, with an algorithm developed inside *μ*-CS, by using the right, chip-specific, annotation library. The resulting preprocessed and annotated data (see Table 1) is structured as a table whose attribute columns contain: (i) the probeset identifier, (ii) the identifier of each sample, (iii) the name of the sequence, e.g. the gene name, (iv) the strand of DNA, (v) the position of start and stop coding region, (vi) the total number of probes, (vii) the cross reference to protein and RNA databases, and (viii) the Gene Ontology annotation. Preprocessed and annotated data can then be analysed with TM4 or eventually with other tools.

**Table 1:** Structure of the output of *μ*-CS.

*probesetid*	*Sample 1*	[...]	*Sample N*	*Gene Name*	*GO*
7896736	3.45	[...]	7.98	ENST00000359325	GO:0032020
7896817	7.91	[...]	9.10	ISG15	GO:0032020

### The structure of preprocessed and annotated data

At the end of a preprocessing job, *μ*-CS generates different output files in a single folder, in order to bring together numerical results, parameter settings and log of activities. Let us suppose that the preprocessing job executed by the user is named myAnalysis. Then the output folder will contain the following files:

myAnalysis-preprocessed-data.txt This file contains the preprocessed data without the annotations. Results are encoded in a simple tabular structure as shown in Table 2. The first column contains the gene identifiers, while the remaining ones contain the expression values of genes in different arrays.

**Table 2:** Example of output of *μ*-CS.

***probeset id***	***1.cel***	***2.cel***
7896736	7.5478	9.2568
7896738	12.5865	18.6561
7896740	3.658	40.475

myAnalysis-preprocessed-annotated-data.txt This file contains the preprocessed and annotated data: for each gene, the annotations provided by the Affymetrix library are reported. The file has a simple tabular structure as shown in Table 3. The first column contains the gene identifiers, while the remaining ones contain the expression values of genes in different arrays. Finally the last one contains the annotation extracted from the Affymetrix annotation library.

**Table 3:** Example of output of *μ*-CS with annotation.

***probeset id***	***1.cel***	***2.cel***	seqname	strand	start	stop
7896736	7.5478	9.2568	chr1	0	42912	44799
7896738	12.5865	18.6561	chr1	0	52878	53750
7896740	3.658	40.475	chr1	0	58954	59871

Affymetrix-Annotations.csv This file contains only the annotations as provided by Affymentrix and is a copy of the Affymetrix annotation library used during the annotation phase. It can be useful to compare data annotated in different times in the case annotation libraries are updated.

myAnalysis-aptlog.txt This file contains the log report generated by the *apt-probeset-summarize *executable. It is useful to detect possible run time errors happening during preprocessing. Future implementations of *μ*-CS will use this file to keep trace of preprocessing parameters that will be encoded in MIAME format.

myAnalysis-mcslog.txt This file contains the log report of *μ*-CS. It is useful to detect possible run time errors of *μ*-CS, happening during preprocessing or annotation.

## Step 5: Analysis

Finally, preprocessed and annotated data can be loaded and analysed by using the functions of TM4.

## Results and Discussion

We presented *μ*-CS (Microarray Cel Files Summarizer), a software tool allowing the semi-automatic summarization and annotation of Affymetrix binary data. The *μ*-CS client allows the summarization and annotation of Affymetrix CEL files datasets by using the proper and most updated Affymetrix libraries. A case study on preprocessing publicly available data is available as supplementary material [see Additional file 1]. In particular, it wraps off-the-shelf preprocessing tools (currently the APT tools are supported) and hides to the user the localization and updating of needed preprocessing and annotation libraries. On the other hand, the *μ*-CS server maintains a repository of metadata about all the entities involved in microarray data preprocessing, among those: chip type, preprocessing and annotation tools and libraries. Moreover, it maintains an updated lists of pointers to relevant preprocessing and annotation libraries that are sent on-demand to the *μ*-CS client via the internet.

Thus, a main contribution of this paper is a software tool that reduces the manual activities needed when preprocessing microarray data and allows to the user to concentrate on the analysis activity hiding the details of libraries localization and updating. In addition, *μ*-CS implements the annotation of data that may improve the analysis process.

Finally, the TM4 plugin version of the *μ*-CS client allows TM4 users to load, preprocess and annotate Affymetrix CEL data that can be further analyzed within the TM4 Suite without wasting time in file management and avoiding possible mistakes due to manual summarization and annotation. By using the *μ*-CS plugin version, TM4 users do not need to know details about preprocessing tools nor need to download and maintain their most recently updated versions, but can easily concentrate on analysis by learning and using TM4 only.

## Comparison with other tools

**Figure 8:**
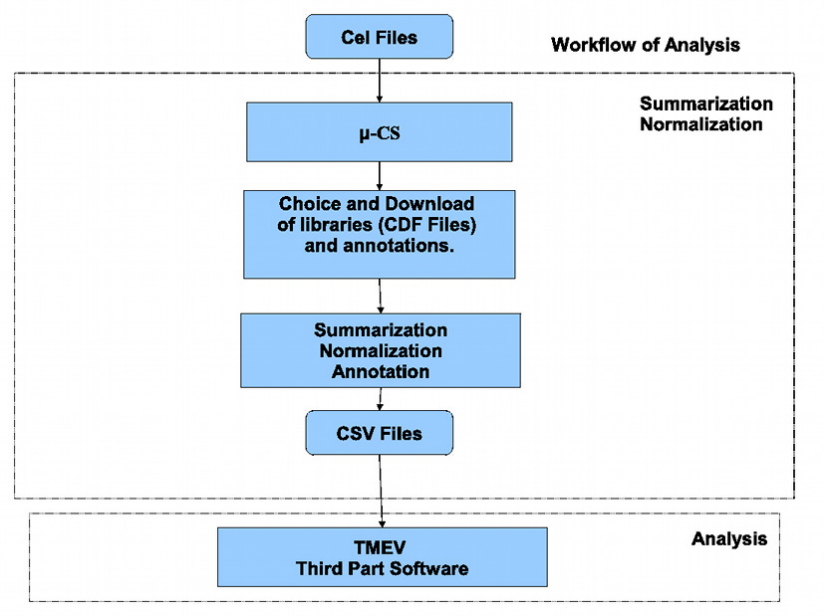
**Workflow of Analysis in *μ*-CS.** A typical workflow of analysis for array data.

In the following we compare, respectively, the use of *μ*-CS with respect to the use of other preprocessing tools, among those: dChip, RMAExpress, Expression Console, easyExon and Taverna workflows. To compare *μ*-CS with the other tools we refer to the workflow of analysis depicted in Figure 8.

## Comparison with dChip

dChip is a standalone program for summarizing Affymetrix Gene expression arrays that is available only for the Windows platform. Compared to *μ*-CS, dChip:

• does not perform automatically the download of libraries (e.g. CDF files);

• performs the Model Based Intensity normalization only;

• does not perform the annotation of files;

• offers both preprocessing and analysis;

• is available only for Windows platform.

**Figure 9:**
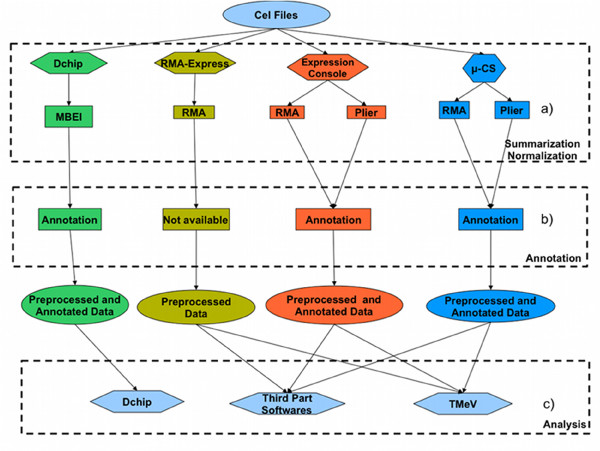
**Comparison with respect to dChip, RMAExpress, and Expression Console**. Each column represents the flow of information when using respectively dChip, RMAExpress, Expression Console, and *μ*-CS. At summarization-normalization layer, label a) indicates which preprocessing algorithms can be chosen. dChip and RMAExpress support only one algorithm, while the other tools include more algorithms, e.g. RMA and Plier. At the annotation layer, label b), all the tools, except for RMAExpress, supports annotation. dChip support annotation using user provided files, while Expression Console and *μ*-CS automatically download annotation files. Thus RMAExpress produces only preprocessed data, while the other tools produce preprocessed and annotated data. Finally at the analysis layer, label c), we note that data provided by dChip can only be analysed with dChip itself, while data provided by the other tools can be analysed by third parts softwares, and in particular by TMeV.

Figure 9 depicts the advantages of using *μ*-CS against dChip. Users that would process expression CEL files with dChip have to manually download libraries, then perform the unique summarization method, and finally need to manually download annotation libraries.

## Comparison with RMAExpress

RMAExpress is a standalone program for summarizing Affymetrix gene expression data that is available for the Windows platform and may be compiled for the Linux platform. Compared to *μ*-CS, it presents some main drawbacks:

• it does not perform automatically the download of libraries (e.g. CDF files);

• it performs only the RMA normalization;

• it does not perform the annotation of files;

• it must be compiled for running on the Linux platform.

Figure 9 depicts the advantages of using *μ*-CS against RMAExpress. Users that would preprocess expression CEL files with RMAExpress have to manually download libraries, then perform the unique summarization method (RMA), and finally need to manually download the annotation libraries and annotate files.

## Comparison with Expression Console

Expression Console [19] is a software provided by Affymetrix that supports probe set summarization of binary CEL files for all the expression arrays. It includes both summarization and quality control algorithms.

Compared to *μ*-CS Expression Console presents some main drawbacks:

• it is not extensible for the preprocessing of multivendor datasets;

• it is not available for Linux platforms.

On the other hand, the current version of *μ*-CS lacks in quality control capabilities compared to Expression Console. Figure 9 compares *μ*-CS against Expression Console.

## Comparison with easyExon

**Figure 10:**
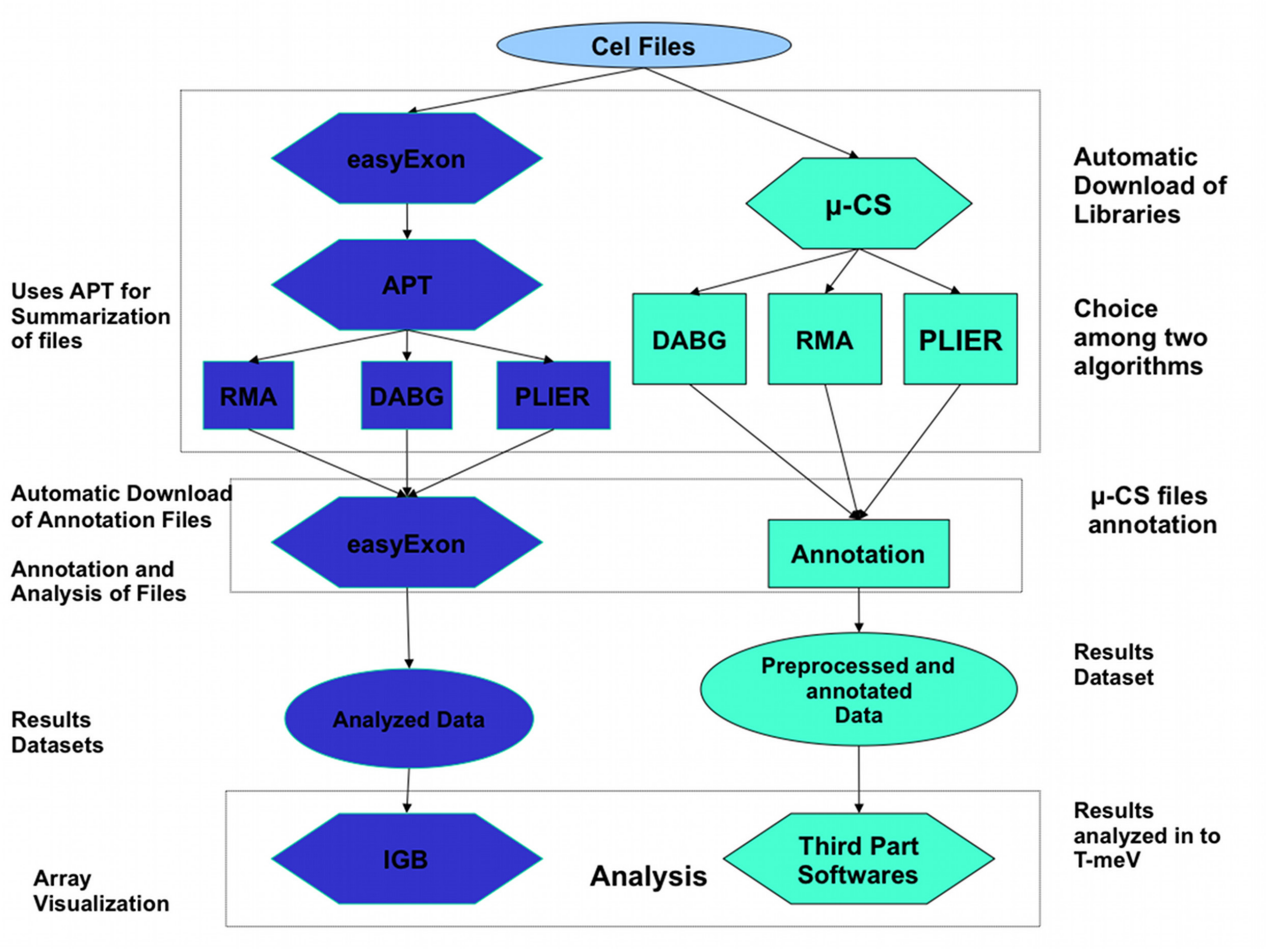
**Comparison with respect to easyExon**. Comparison of easyExon and *μ*-CS with respect to a workflow of analysis

easyExon [20] is an integrated pipeline for preprocessing and analysis of exon array data. easyExon can receive as input either summarized files or binary CEL files that is able to manage by calling Affymetrix APT tools. easyExon implements some main algorithms for analysis of alternative splicing events and it is integrated with external software tools, such as the APT and the Integrate Genome Browser (IGB) for, respectively, the preprocessing and the biological interpretation of data. Compared to *μ*-CS (see Figure 10) it presents the following main differences:

• *μ*-CS has a broaden range of applications in terms of managed chips, in fact easyExon focuses only on exon array data;

• *μ*-CS focuses only on the preprocessing, while easyExon offer analysis capabilities;

• *μ*-CS can summarize even exon arrays for detecting alternative splicing by using the DABG algorithm integrated into APT and selectable through the *μ*-CS interface.

## Comparison with Taverna workflows

There exist other approaches for preprocessing microarray data that employ distributed architectures based on Web Services and SOAP messaging. For instance, the work [21] presents a workflow of identification of differentially expressed genes by using different Web Services available into the Taverna platform. The proposed workflow aims to retrieve microarray data stored into an ad hoc developed database and to process them by using the R statistical package deployed as a Taverna service. Compared to this solution, *μ*-CS presents the advantage that it does not require the movement of data from the analysis laboratory to an external service (i.e. the maxdLoad2 database in the case of that workflow) and the installation and the usage of the Taverna platform. On the other hand, it does not perform all the analysis steps that are available in such a workflow, for instance the identification of differentially expressed genes.

## Conclusions

The study of gene expression data is nowadays an important field of research strategy. Microarrays enable the investigation of such reality by using chips that are able to scan the whole genome, e.g the Affymetrix Human Gene 1.0 ST array.

The preprocessing of microarray data is an important task that is often: i) performed manually, i.e. by using proprietary tools and related libraries and taking care of file management; and ii) conducted outside of common analysis platforms, such as TM4, thus limiting the throughput of the analysis pipeline and augmenting the probability of errors due to manual activities.

So, the automation of the preprocessing tasks and their integration into main analysis platforms may improve the entire microarray pipeline, by reducing the manual interventions on data (e.g. copy and paste of files from preprocessing to analysis tools) and by guaranteing the usage of the most recent libraries made available by microarray vendors.

In this paper we proposed *μ*-CS, a client/server tool that natively reads and preprocesses Affymetrix microarray data by wrapping existing preprocessing tools and by providing the most updated summarization and annotation libraries. The *μ*-CS server, that adopts a web services architecture, maintains an updated list of libraries available on the Affymetrix repositories. The *μ*-CS client, made available both as a standalone tool and as a TM4 plugin, allows the integrated preprocessing and analysis of Affymetrix data by using just one platform.

The existing version of *μ*-CS has been tested using microarray data publicly available on the Affymetrix web site. We also compared *μ*-CS with respect to the main preprocessing tools, considering qualitative aspects such as: (i) automatic update of summarization libraries, (ii) automatic annotation of gene expression data, (iii) independence from the operating system, (iv) integration with TM4. The summarization tools considered in the comparison are: dChip, RMAExpress, Expression Console, easyExon, and webservices available on Taverna. dChip does not implements all the summarization algorithms provided by *μ*-CS and requires the manual installation of libraries. RMAExpress supports the summarization of CEL files but compared to *μ*-CS presents four main drawbacks: (i) it does not provide the automatic updating of the needed libraries, (ii) it implements only the RMA algorithm, (iii) it does no provide annotation, and (iv) it is available only for Windows operating system. easyExon is a Java tool that is able to implement alternative splicing events but with respect to *μ*-CS is not easily extensible to other chips. Finally Web Services available on Taverna require more expertise to be used. Moreover APT Tools offer only a command line interface and do not automatize the management of libraries. Future work will regard two main directions: the generalisation of the preprocessing steps in order to make possible the management of many microarray data, e.g. Illumina Bead Array, and the implementation of the preprocessing tasks as a service.

## Availability and requirements

• Project name: *μ*-CS

• Project home page: http://bioingegneria.unicz.it/m-cs.

• Operating system(s): *μ*-CS tool is available for Windows and Linux operating systems.

• Programming language: Java

• Other requirements: Java 1.4.1 Runtime or higher.

• License: GNU GPL.

• Any restrictions to use by non-academics: The software is for academic purposes only.

## Authors' contributions

MC and PHG conceived the idea and designed the proposed software tool. All authors read and approved the final manuscript.

## Supplementary Material

Additional file 1 **S1**. File contains the discussion of some case studies and a deeper comparison with respect to other softwares. Click here for file
