# Supplementary material for: μ-CS: An extension of the TM4 platform to manage Affymetrix binary data
Source: BMC Bioinformatics. 2010 Jun 10;11:315. doi: 10.1186/1471-2105-11-315 (PMC2907348; doi:10.1186/1471-2105-11-315)
Supplement: Additional file 1 — S1. File contains the discussion of some case studies and a deeper comparison with respect to other softwares. [file 1471-2105-11-315-S1.PDF]

μ-CS: An extension of the TM4 platform to manage Affymetrix binary data Supplementary Material

Mario Cannataro and Pietro Hiram Guzzi

June 18, 2010

Abstract

1 Using $\mu - CS$

The first output of a microarray experiment on an Affymetrix platform is an image where pixels intensities are related to gene expressions values. Images are usually encoded into binary cel files that use a proprietary format and are not automatically readable from existing analysis platforms. Existing freely available analysis platforms, such as the TM4 platform are not able to directly manage the Affymetrix CEL files, so the user has to perform some steps manually employing external tools such as the Affymetrix Expression console or the Affymetrix Power Tools, or third part software such as RMAExpress. Main phases of the preprocessing of Affymetrix arrays are :*i*) normalization, (*ii*) summarization, and (*iii*) annotation. normalization consists of reducing the bias among chips and within different regions of the same chip, aiming at removing non-biological variability within a dataset. summarization combines multiple preprocessed probe intensities to a single expression value. Annotation associates to each probe its known annotations such as Gene Symbol or Gene Ontology. Each step of preprocessing requires appropriates libraries that are provided by Affymetrix and that are periodically updated.

The μ-CS sits in the middle of this analysis process and offer preprocessing services, as depicted in Figure 1.

μ-CS is a client/server tool that natively reads and preprocesses Affymetrix microarray data by wrapping existing preprocessing tools and by providing the most updated summarization and annotation libraries. The μ-CS server, that adopts a web services architecture, maintains an updated list of libraries available on the Affymetrix repositories. The μ-CS client is available both as a standalone tool and as a TM4 plugin, allowing the integrated preprocessing and analysis of Affymetrix data using just one platform.

The user that would preprocess array data by using μ-CS has to do the following steps:

Step 1: Installing/updating libraries . Initially user has to install the libraries for the chip or can check the availability of updates for the already

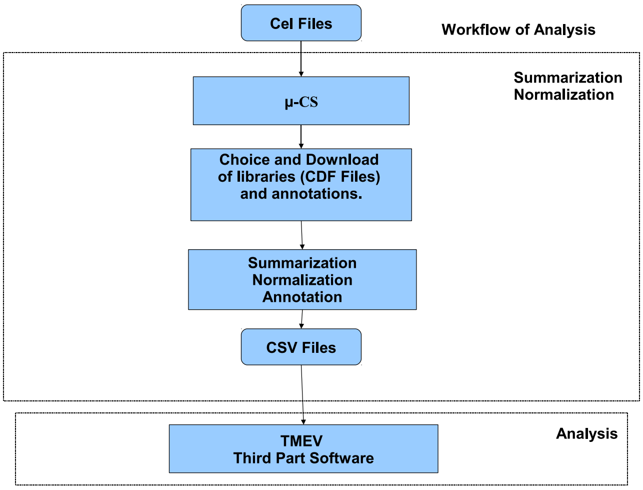

installed ones. This operation can be simply done by selecting the name of the chip, then μ-CS will retrieve and install the libraries.

Step 2: Loading dataset and Selection of Chip Libraries . Then user can load the dataset through the GUI and in a parallel way can select the chip type, so μ-CS can use the proper libraries.

Step 3: APT parameters setting . After the loading user has to select main parameters of analysis. In case of Standard analysis he has to select: Summarization algorithm and Normalization method. Alternatively, in Advanced Analysis Mode he can write the command line for APT.

Step 4: Preprocessing and annotation . μ-CS reads the input binary files and invokes the APT executable by using the user's specified parameters. When summarization and annotation are completed, μ-CS writes the results file for subsequent analysis.

Step 5: Analysis . Preprocessed and annotated data can be analysed by using TM4 or other analysis tools.

2 Case Studies on Affymetrix Chips

2.1 Preprocessing of Affymetrix Rat Gene1.0st

This Section shows the functionalities of μ-CS through a case study regarding the preprocessing of Affymetrix binary files.
Dataset.
The analysed dataset is a freely available dataset produced by using the RatGene 1.0 ST chip and downloadable at the Affymetrix website [1]. Data set contains a collection of 10 tissues of Rat, and each tissue has three biological replicates. The RNA samples are from a commercial source. Each tissue has three biological replicates.

[1] $http://www.affymetrix.com/support/technical/sample\_data/datasets.affx$

- **Step 1: Installing/updating libraries**: The loaded dataset consists of ten arrays of RatGene 1.0 st. User, after launching the client has to require the installation of libraries for this array. The libraries are selected through the μ-CS client that then checks their availability on the local database.

- **Step 2: Loading dataset and Selection of Chip Libraries**: The loaded dataset consists of ten arrays of RatGene 1.0 st.

- **Step 3: APT parameters setting**: the user chooses the main parameters of the APT preprocessing tool, in particular the following settings are performed Summarization=RMA, sketch=Yes;

- **Step 4: Preprocessing and annotation**: μ-CS reads the input binary files and invokes the APT executable by using the user's specified parameters. When summarization and annotation are completed, μ-CS writes the results file for subsequent analysis.

- **Step 5: Analysis**: preprocessed and annotated data can be analyzed by using TM4 or other analysis tools.

Results of preprocessing are available at the project web site (`http://bioingegneria.unicz.it/~guzzi/m-cs`).

2.2 Preprocessing of 3IVT Arrays

This Section shows the functionalities of μ-CS through a case study regarding the preprocessing of Affymetrix binary files based on a freely available datasets of Human Genome U133 Plus 2.0 dataset produced by Affymetrix.
Dataset.

The analysed dataset is a freely available dataset produced by using the Human Genome U133 Plus 2.0 and downloadable at the Affymetrix website. Data set contains a collection of 11 tissues of Human, and each tissue has three biological replicates. The RNA samples are from a commercial source. Each tissue has three biological replicates.

- **Step 1: Installing/updating libraries**: The loaded dataset consists of all the arrays of Human Genome U133 Plus 2.0. User, after launching the client has to require the installation of libraries for this array. The libraries are selected through the μ-CS client that then checks their availability on the local database.

- **Step 2: Loading dataset and Selection of Chip Libraries**: The loaded dataset consists of all the arrays of Human Genome U133 Plus 2.0 .

- **Step 3: APT parameters setting**: the user chooses the main parameters of the APT preprocessing tool, in particular the following settings are performed Summarization=RMA, sketch=Yes;

- **Step 4: Preprocessing and annotation**: μ-CS reads the input binary files and invokes the APT executable by using the user's specified parameters. When summarization and annotation are completed, μ-CS writes the results file for subsequent analysis.

- **Step 5: Analysis**: preprocessed and annotated data can be analyzed by using TM4 or other analysis tools.

Results of preprocessing are available at the project web site.

2.3 Preprocessing of Human Exon 1.0 ST Data

This Section shows the functionalities of μ-CS through a case study on Affymetrix binary files based on a freely available datasets of Human Exon 1.0 ST Data dataset produced by Affymetrix.

Recently the interest of researcher has focused on the determination of Alternative Splicing events (AS), i.e. the process that causes the production of multiple forms of a protein from the same gene. A gene is composed by a number of coding regions, called exons, that together participate in the protein synthesis. Nevertheless, Alternative Splicing causes the activation or the inhibition of a subset of exons causing the production of different forms, called isoforms of the same protein. Thus researchers needed of a platform that interrogates not only the genes but all the coding regions of a single gene. Affymetrix Exon chips for Human Mouse, and Rat enable such investigation Exon chips can be used to interrogates the whole gene, **gene-level analysis**, or all the exons, **exon-level analysis**. The analysis of exon arrays involves the following steps as depicted in Figure 1: (i) Normalization, (ii) Summarization, (iii) Filtering to remove poorly performing probesetsDetection calls, and (iv) statistical analysis, i.e. detection of differentially expressed exons. The step (i) and (ii) can be made by using classical normalization and summarization algorithms, e.g. RMA or PLIER, while the third step has to be performed by using an ad hoc algorithm, called Detection Above Background (DABG), that increases the data quality by evaluating the reliability of the measure associated to each exon and removing those that are unreliable.

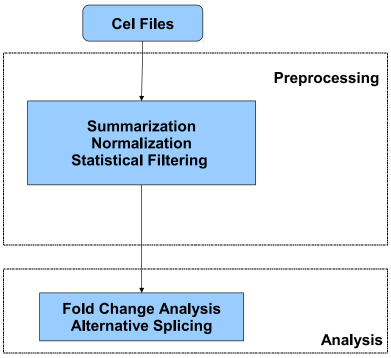

Figure 1: Analysis of Exon Arrays

Exon arrays can be easily managed into μ-CS by selecting the right libraries.

Then μ-CS is able to perform both summarization and detection of background. Results of preprocessing are available at μ-CS the project web site.

3 Annotation of Affymetrix Files

The periodicity used by Affymetrix for updating its annotations is not known a priori because it depends mainly on the kind of chip, and on the analyzed species, e.g. human or rat or mouse. Thus the user cannot foresee when a new version of the annotation libraries will be available. To provide an estimate of the growth rate of such libraries we considered the Affymetrix releases of the annotation of the human genome. Since 2007, Affymetrix built 9 releases of annotation for Human Genome (all these files are available for download on the Affymetrix website http://www.affymetrix.com/analysis/downloads/ after a free registration). Table 1 reports, for each released version, the release date, the library name, and the dimension in KBs, and Figure 2 depicts the growth of these files.

Table 1: Growth of Annotations Files for Human Genome

**Release**	**Date**	**Name**	**Size (Kbs)**
22	28/03/2007	*HuGene1_0stv1.na22.hg18.transcript.csv*	12204
23	15/03/2007	*HuGene1_0stv1.na23.hg18.transcript.csv*	11436
24	11/12/2007	*HuGene1_0stv1.na24.hg18.transcript.csv*	8549
25	28/02/2008	*HuGene1_0stv1.na25.hg18.transcript.csv*	10324
26	21/07/2008	*HuGene1_0stv1.na26.hg18.transcript.csv*	11458
27	01/12/2008	*HuGene1_0stv1.na27.hg18.transcript.csv*	10421
28	16/03/2009	*HuGene1_0stv1.na28.hg18.transcript.csv*	12499
29	13/07/2009	*HuGene1_0stv1.na29.hg18.transcript.csv*	14418
30	16/11/2009	*HuGene1_0stv1.na30.hg19.transcript.csv*	18405

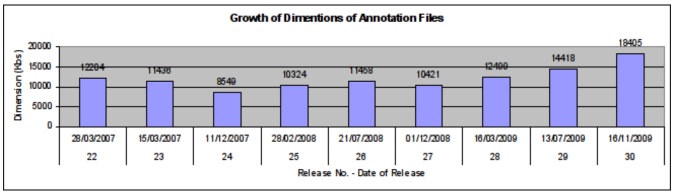

Figure 2: Growth of Dimensions of Human Gene Annotation Files

For the MouseGene 1.0 st, Affymetrix built these releases as shown in the Table 2 (all these files are available for download on the Affymetrix website after a free registration).

Figure 3 depicts the growth of these files.

Table 2: Growth of Annotations Files for Mouse Genome

**Release**	**Date**	**Name**	**Size (Kbs**
24	2007/11	$MoGene1\_0stv1.na24.mm9.transcript.csv$	6259
25	2008/03	$MoGene1\_0stv1.na25.mm9.transcript.csv$	7410
26	21/07/2008	$MoGene1\_0stv1.na26.mm9.transcript.csv$	10154
27	01/12/2008	$MoGene1\_0stv1.na27.mm9.transcript.csv$	10046
28	16/03/2009	$MoGene1\_0stv1.na28.mm9.transcript.csv$	10044
29	13/07/2009	$MoGene1\_0stv1.na29.mm9.transcript.csv$	11732
30	16/11/2009	$MoGene1\_0stv1.na30.mm9.transcript.csv$	11276

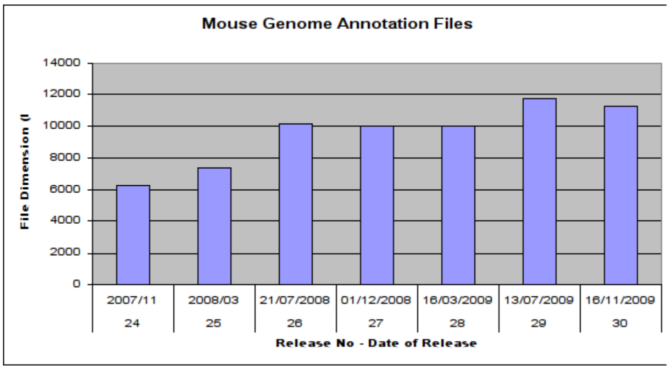

Figure 3: Growth of Dimensions of Mouse Gene Annotation Files
